# RepFluo, a Fast Fluorescent In Vitro Assay of Cas9 Activity Exploiting Melting Curve Analysis

**DOI:** 10.1021/acsomega.5c02066

**Published:** 2025-11-09

**Authors:** Filippo Fronza, Roberto Verardo, Claudio Schneider

**Affiliations:** † Dipartimento di Medicina (DMED), 9316 University of Udine, Piazzale Kolbe 1, Udine 33100, Italy; ‡ Consorzio Interuniversitario per le Biotechnologie, 18471 LNCIB Laboratorio Nazionale CIB, BIC Incubatori, Via Flavia 23/1, Trieste 34148, Italy; § AREA Science Park, Patriciano 99, Trieste 34149, Italy

## Abstract

Demand for less labor-intensive in vitro assays of the activity of CRISPR/Cas proteins is rising to extend the potential applications of CRISPR in the field of diagnostics. RNA guided DNA endonucleases of the Cas family generate double-strand breaks in the target DNA, which results in two shorter DNA fragments. We hypothesized that this cleavage event could be studied using melting curve analysis, and using SpyCas9, we demonstrate that it is possible to evaluate the activity of Cas proteins by measuring the melting curves of their products. We present here a novel assay for the in vitro activity of Cas9 that exploits melting curve analysis (MCA) to be fast, inexpensive, and widely accessible. The assay can, in fact, be performed with readily available components—in its simplest form a real-time thermal cycler and an intercalating dye (SYBR Green I)—and produces reliable results with a run-time of 15 min. It does not require external intervention to stop the reaction, which is done by thermal denaturation of the protein directly in the thermal cycler machine. The described advantages, combined with the provided data analysis package, make the assay robust and amenable to high-throughput applications. To increase the accessibility of our assay, we provided an R package that simplifies the analytical process.

## Graphical abstract

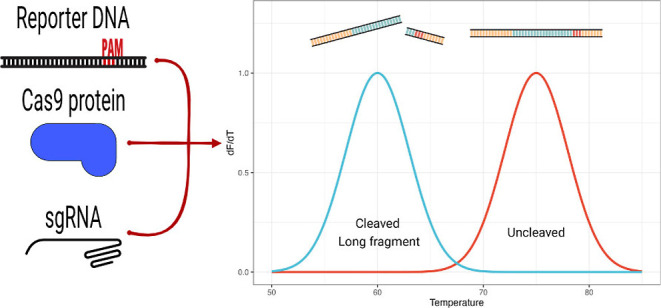

## 1 Introduction

The exploitation of the clustered regularly interspaced short palindromic repeats (CRISPR)^1,2^ is based on the activity of CRISPR-associated nucleases, with type II CRISPR enzyme Cas9 being the most well-studied example.

CRISPR-based technologies have been focused on in vivo or ex vivo applications, through the development of many techniques that streamline its usage with cellular and animal models,^3,4^ with promising results in the treatment of genetic disorders, neurodegenerative disorders, and cancer.^5^

More recently, the emergence of SARS-CoV-2 has presented an opportunity for the in vitro use of CRISPR, sparking the development of innovative diagnostic techniques such as SHERLOCK, DETECT,^6^ and LEOPARD,^7^ leading to the birth of the CRISPR-based diagnostics (CRISPR-dx).^8^

### 1.1 In Vitro Assay

Current assays monitoring in vitro Cas9 activity either require specialized equipment^9^ or are time-consuming.^10^

Electrophoretic separation of the cleavage products either on agarose or polyacrylamide gels remains the gold standard for Cas9 cleavage assays.^1,11^ These approaches, which distinguish the shorter dsDNA fragments that are generated by the cleavage from the uncleaved target based on their size, are, however, both time-consuming and limited in throughput.

We hypothesized that it would be possible to follow the activity of Cas9 using melting curve analysis (MCA) given that these DNA fragments, shorter in length with respect to the uncleaved target DNA, have a lower melting temperature (Scheme 1).

**Scheme 1:**
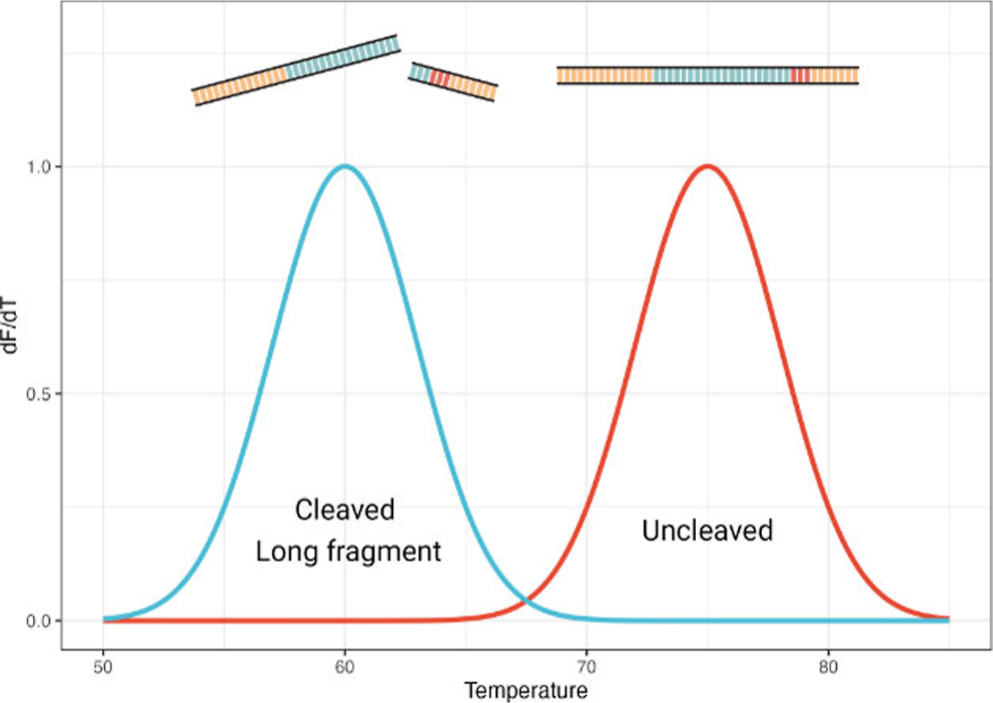
Hypothesized Change in Melting Temperature after Cas9 Cleavage^a^

a Because the cleaved fragments have a lower melting temperature than the original uncleaved target DNA, it should be possible to discriminate the two using melting curve analysis.

Here, we present a novel in vitro assay for the detection of Cas9 cleavage activity based on melting curve analysis^12,13^ of reporter DNA containing the target for Cas9. When the reporter is combined with the Cas9 protein and the appropriate guide RNA (RNP), cleavage of the DNA fragment changes the melting temperature and melting peak profile. The assay is designed to be simple, fast, versatile, and amenable to high-throughput applications and is performed in a one-pot reaction using a real-time thermal cycler for precise control of temperature and reaction times (Scheme 2). Minimal sample manipulation is required once the reaction is started by combining the Cas9 RNP with the reporter DNA.

**Scheme 2:**
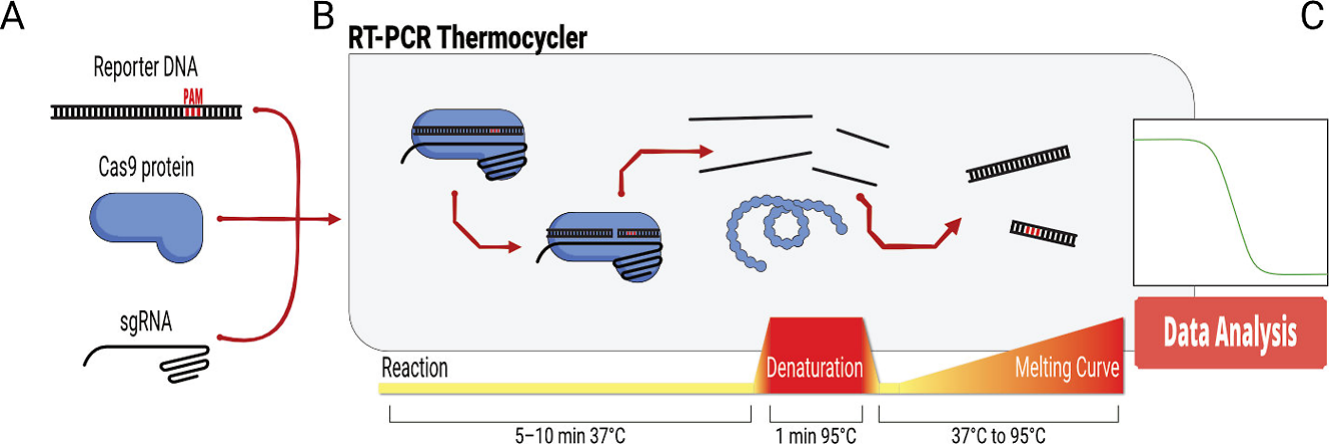
Schematic Description of the Assay: (A) a Reporter DNA Is Mixed Together with the Cas9 Protein and sgRNA. (B) The Reaction Is Then Loaded into a Real-Time Thermal Cycler, Where It Is Carried On at a Controlled Temperature. After an Incubation Step, the Ribonucleoprotein Complex Is Denatured at 95 °C to Release Fragments of the Reporter DNA. Finally, Readout Is Performed via Melting Curve, and (C) the Results Are Evaluated Computationally to Estimate Protein Activity

## 2 Methods

### 2.1 SpyCas9 Protein and sgRNA

SpyCas9 protein was obtained from IDT (Alt-R Cas9 Nuclease v3) or purified in-house as His-MBP-SpyCas9 ^14^ (Supporting Information Figure S2). Immediately before use, the protein was diluted in PBS (Merk, Sigma-Aldrich).

sgRNAs were designed, amplified, in vitro transcribed, and purified using the Precision gRNA Synthesis Kit (ThermoFisher Scientific, Invitrogen) as described in Supporting Information

Scramble control sgRNA (Scr) was amplified before in vitro transcription from pUC57-sgRNA expression vector, a gift from Xingxu Huang (Addgene plasmid # 51132; http://n2t.net/addgene:51132 ; RRID:Addgene_51132)^15^

### 2.2 Reporter DNA Design

To be able to measure the change in melting curves before and after cleavage, we designed short synthetic dsDNA oligonucleotides (IDT) containing a target sequence—as reporters of Cas9 activity.

These were between 41*bp* and 44*bp* in length, with expected melting temperatures between 60 and 80 °C. Each reporter contains a single target sequence, originally from either the RPP30 subunit of RNase P—a housekeeper gene—or the E gene from the SARS-CoV-2 virus.

We tested several designs that offered different readout options, and here we present the two most promising ones.

The first analyzed design (*Fluo*-*Quencher RPP30 alias FQ*-*RPP30*) has a fluorophore and a quencher covalently attached to the reporter nontarget strand (NTS) and an elongated target strand (Scheme 3A). The readout for this design is based on the interaction between the fluorophore and quencher during the melting curve process.

**Scheme 3:**
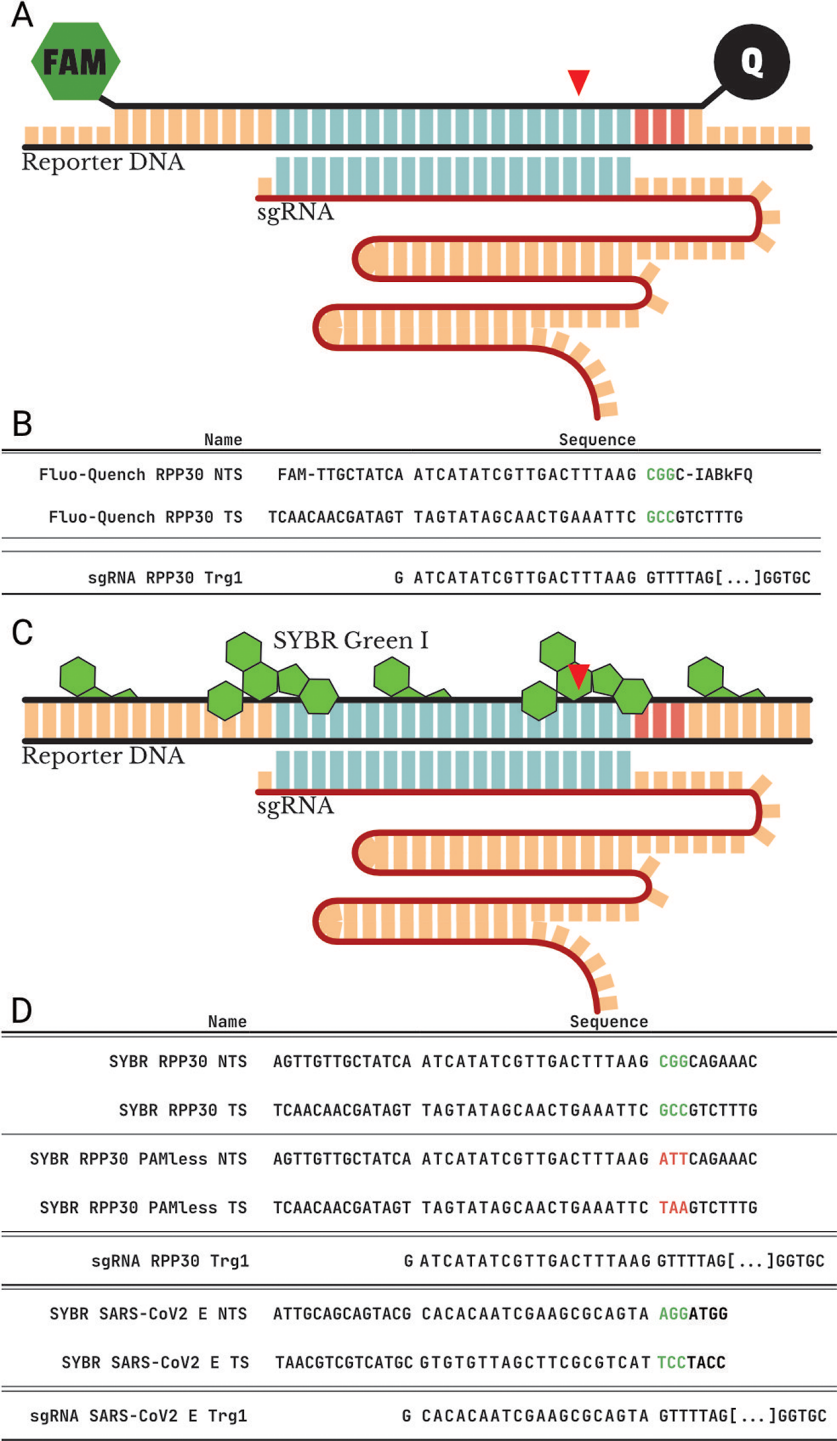
Representation of the Structure of the Presented Reporters, with Matching sgRNA. (A) Fluorophore Quencher Reporter with 5,6-FAM at the 5′ of Its Nontarget Strand and Iowa-Balck Quencher (IDT) at Its 3′end. (B) SYBR Green I-Based Reporter: Fluorescence Is Provided by the Intercalating Dye. In Both Representations, the Red Arrow Marks the Position of the Double-Strand Break That Is Produced by Cas9; in Pale Blue, the Target Sequence Is Marked; and in Pale Red the PAM. (D,E) Sequences of the Reporters and sgRNAs

The second design (*SYBR RPP30*, *SYBR RPP30 PAMless*, and *SYBR SARS*-*CoV2 E*) is a simple dsDNA fragment with strands of equal length (Scheme 3C). The readout for this reporter is provided by an intercalating dye, SYBR Green I, which is added to the reaction.

### 2.3 In Vitro Reporter DNA Cleavage Assay

Reactions were prepared by mixing reporter DNA (25 nM final concentration) together with SpyCas9 protein and sgRNA—in a ratio of 1:3:3.6—in rCutSmart (NEB) buffer to a final volume of 40μL. When using SYBR Green-based reporter, a final concentration of 500nM SYBR Green I (Jena Bioscience) was also added. Samples were assembled in a PCR microplate, mixed thoroughly, vortexed, and spun, then loaded into a real-time thermal cycler, without preforming any preincubation step of protein and sgRNA.

Two different real-time thermal cyclers were used for the experiments: either a BioRad CFX 96 or a Thermo Fisher Scientific QuantStudio Q3. Data import procedures are specified in Supporting Information Section S4. No significant difference in quality was observed between the two instruments.

Samples were incubated at 37 °C for 5 to 10 min, after which the reaction was stopped by raising the temperature to 95 °C for 1 min to inactivate the enzyme. The melting curve was then recorded from 37 to 95 °C as readout (Scheme 2), with reads taken every 0.2 to 0.5 °C at the fastest ramping speed available depending on the real-time thermal cycler instrument used.^16^

### 2.4 Data Analysis and Gaussian Mixture Modeling

Raw data were min–max normalized, smoothed,^17^ then baseline corrected.^18^

The signal *S*(*x*) was then modeled as a Gaussian mixture model (GMM)^19^ of skewed generalized normal distributions^20^ scaled to a factor *A*.

1 $$NSGN\;\left(x\right)=\frac{\beta }{2^{1+1/\beta }\Gamma \left(1/\beta \right)\sigma }e^{-{\left|x-\mu \right|}^{\beta }/2\sigma^{\beta }{\left[1+rsign\left(x-\mu \right)\right]}^{\beta }}$$

where *r* is a skewness parameter between −1 and 1, Γ is the gamma function, and β is a shape parameter.

When scaled by the factor *A*

2 $$A=\alpha \frac{2^{1+1/\beta }\Gamma \left(1/\beta \right)\sigma }{\beta }$$

the max.arg of *A*·*NSGN* is equal to α.

Thus, *S* can be defined as a sum of Gaussians, plus an error ϵ.

3 $$S=\sum_{i=1}^{n}\left(A_{i}NSGN_{i}\right)+\epsilon$$

where *n* is the number of Gaussians in the model, and their height, α_*i*_, is equal to the value of *S*(μ_*i*_). We take advantage of this property in the modeling process.

At each iteration of the modeling process, we assign max­(*S*) to α and *arg *max_*x*_(*S*(*x*)) to μ. Then σ, *r*, and β are optimized to minimize the L2-norm ∥*S* – *A*(α)*NSGN*(μ, σ, *r*, and β)∥_2_

The fitted values are subtracted from the signal, and the process is repeated until max­(*S*) falls below the noise threshold.

While the model obtained is a faithful representation of the signal, it tells us little about the probability of our reporter being denatured at a certain temperature. To transform our model into a probability density function, we take the cumulative function of *NSGN*

4 $$CDF^{ngsk}\left(x\right)=\{\begin{array}{ll} \frac{1-r}{2}\left[1-\gamma \left(\frac{{\left(\mu -x\right)}^{\beta }}{2\sigma^{\beta }{\left(1-r\right)}^{\beta }};\frac{1}{\beta }\right)\right], & x<\mu \\ \frac{1-r}{2}+\frac{1+r}{2}\gamma \left(\frac{{\left(x-\mu \right)}^{\beta }}{2\sigma^{\beta }{\left(1+r\right)}^{\beta }};\frac{1}{\beta }\right), & x\geq \mu \end{array}$$

which is monotonic and right-continuous. Then we multiply its summation with a constant ρ at the limits of *x* ∞ and −∞ to satisfy the following conditions

5 $$\lim_{x\to \infty }\sum_{i=1}^{n}\rho A_{i}CDF_{i}^{nsgn}=1$$

6 $$\lim_{x\to -\infty }\sum_{i=1}^{n}\rho A_{i}CDF_{i}^{nsgn}=0$$

Considering that the limits of the CDF are lim_*x*→–∞_CDF^*nsgn*^ = 0 and lim_*x*→∞_CDF^*nsgn*^ = 1, we can solve for $\rho =\frac{1}{\sum_{i=1}^{n}A_{i}}$ and use it to scale each Gaussian such that the model as a whole becomes a probability density function.

Since the scale of each Gaussian is controlled by the α factor of *A* (eq 2), we can calculate a new α for each curve, the related probability density function (PDF) and cumulative distribution function (CDF).

7 $$\alpha^{new}=\rho \alpha^{old}=\frac{\alpha^{old}}{\sum_{i=1}^{n}A_{i}}$$

8 $$PDF^{S}=\sum_{i=1}^{n}A{\left(\alpha_{i}^{new}\right)}_{i}NSGN_{i}$$

9 $$CDF^{S}=\sum_{i=1}^{n}A{\left(\alpha_{i}^{new}\right)}_{i}CDF_{i}^{nsgn}$$

Under the assumption that at 37 °C all the reporter DNA is in a double-strand state and at 95 °C is fully denatured, the value of CDF^S^ at a temperature *T* is the probability that the reporter is denatured at a temperature equal to or lower than *T*.

### 2.5 Statistical Analysis

For each experiment, we evaluated the value of CDF^S^ at the temperature where the standard deviation of the CDF^S^ of all samples was the highest. We call this value cumulative at max variance (CMV).

Each group was tested for normality with the Shapiro–Wilk test. If the assumption of normality was met, samples were tested pairwise using the *t*-test; otherwise, the Wilcoxon–Mann–Whitney test was used.

### 2.6 PAGE

All reactions prepared for polyacrylamide gel electrophoresis were scaled up to 100 nM of reporter DNA while keeping the same ratio of reporter:Cas9:sgRNA of 1:3:3.6, maintaining a final volume to 40 μL.

Denaturing 12% urea-PAGE^21^ was used to check the cleavage of each strand of the Fluo-Quench reporter DNA. After the electrophoretic run, the gel was fixed and stained in EtBr solution (0.5 mg/mL EtBr, 1× TBE; Merk) for 30 min, then washed three times in water. Pictures were taken before and after staining.

Native 12% page^22^ was used to check the cleavage of SYBR Green-based reporter DNAs. If bands were not clearly visible with SYBR Green dye, then gels were stained in EtBr as indicated.

All pictures were taken using FireReaderV10 (Uvitec) and level adjusted using Affinity Photo (Serif) with a 5p× Dust and Scratches filter applied. False colors were supplied as a gradient map.

### 2.7 Quantitative Association

The relationship between the melting curves observed and the amount of cleaved product was evaluated by using synthetic oligonucleotides equivalent to the products of the reaction. We will call them pseudocleaved reporter DNA fragments.

For the fluorophore-quencher reporter *FQ*-*RPP30,* these are a dsDNA oligonucleotide going from the star of the uncleaved reporter to the cleavage site at PAM-3, with the fluorophore at the 5′ end of the NTS, and a second oligonucleotide going from PAM-3 to the end of the sequence, with the quencher at the 3′ of the NTS.

Similarly, the two pseudocleave fragments for the SYBR Green-based reporter *SYBR RPP30* go from the start of the sequence to the PAM-3 cleavage site and from the PAM-3 cleavage site to the end of the sequence.

Uncleaved reporter DNA and pseudocleaved fragments were mixed at different ratios (0:7, 1:6, 2:5, 3:4, 4:3, 5:2, 6:1, and 7:0) to simulate a varied amount of cleavage products. The mixtures were prepared to a final concentration equivalent to 25nM of reporter DNA—accounting for the fact that the two pseudocleaved fragments are treated as a single object—in rCutSmart buffer and to a final volume of 40μL. They were then incubated at 37 °C for 5 min and denatured at 95 °C for 1 min, and a melting curve was recorded from 37 to 95 °C, as for the assay presented above.

### 2.8 Temperature Inactivation

Heat treatment experiments were performed to evaluate if the denaturation step described in the standard assay (1 min at 95 °C) was sufficient to completely inactivate the Cas9 RNP and exclude the possibility that the cleavage reaction continues when the temperature is lowered to start the melting curve.

Reaction mixtures were prepared by combining SpyCas9 and sgRNA in rCutSmart buffer according to the protocol described in the previous section but excluding the reporter DNA, which was introduced after the heat treatment. Samples were then subjected to heat treatment at a temperature between 35 and 60 °C for 1 min. After heat treatment, SYBR Green-based reporter DNA was added to each reaction, and the incubation, denaturation, and melting curve steps of the assays were performed as described above.

Inactivation temperature was evaluated via logistic regression using the equation

10 $$f\left(x\right)=\frac{1}{1+e^{-\left(x-\mu \right)/s}}$$

In order to fit the logistic regression, the addition of a small amount of jittering noise to the heat treatment temperatures (within 2 °C) was necessary.

### 2.9 Evaluation of Reaction Kinetics

To test the reaction kinetics, the assay was repeated at six incubation lengths, by stopping the reaction at different time points: 0 min, 1 min, 2.5 min, 5, 10, and 20 min. To account for the fact that SpyCas9 cleavage reaction may start at room temperature^23^ and achieve more accurate measurements, the incubation time was chronographed from the addition of the reporter DNA to the mixture until the thermal cycler reached 95 °C.

The reaction kinetics were modeled by fitting them to a double exponential eq (eq 11).^24^

11 $$Y=A_{1}\cdot \left(1-e^{-\lambda_{1}\cdot t}\right)+A_{2}\cdot \left(1-e^{-\lambda_{2}\cdot t}\right)+C$$

## 3 Results and Discussion

### 3.1 Processing of MCA Data

Melting curve analysis traditionally serves as a qualitative control in real-time PCR applications, where manufacturer software typically handles data processing with predefined parameters. However, we desired more control over the processing of our assay data to approach a quantitative interpretation of melting curves and assess enzymatic activity, necessitating a more sophisticated analytical pipeline. We developed a custom processing pipeline based on two key assumptions: 1. the amount of reporter DNA is consistent across samples within an experiment; 2. the reporter DNA exists fully in the double-strand state at 37 °C and is fully denatured at 95 °C.

Raw fluorescence data from melting curve experiments exhibit inherent variability in signal intensity, likely due to minor differences in reaction volume and DNA concentration (Figure S1A), with no significant difference in quality observed between the two instruments used (Figure S3).

To enable meaningful comparison between different samples and different instruments, we applied min–max normalization, scaling all curves to the range [0,1] (Figure S1B). While this transformation precludes direct quantification of absolute DNA amounts, it enables reliable comparison of melting profiles between different samples and throughout different experiments. This is a standard practice in high-resolution melting (HRM) analysis.^25^

The derivative of the melting curve signal was obtained using the Savitzky–Golay algorithm,^26^ which combines differentiation and smoothing. To further contain signal noise, we applied a smoothing step before differentiation using Friedman’s Super Smoother^27^ (Supporting Figure S1C,D).

A particular challenge in the evaluation of our assay, specifically for the SYBR Green-based assay, was the presence of a shifting baseline that manifests as a shoulder in the left region of the melting curve. Standard melting curve analysis typically ignores this artifact but can significantly impact quantitative interpretation. We addressed this through the BEADS baseline correction algorithm,^18^ originally developed for chromatography applications, which effectively handles asymmetric signals (Supporting Figure S1E).

The final challenge to address was the evaluation of the melting peaks by quantifying the difference between the melting curves of the samples into a single representative value and evaluating the probability that the reporter DNA was denatured at a certain temperature. A naïve numerical approach would be to normalize the height of the tallest peak and calculate a numerical cumulative, which, however, introduces a bias if the melting curve is composed of multiple peaks. In fact, the ratio between the peaks remains constant, while the total volume under the curve does not. A second approach would be to first integrate the peaks, then normalize the data, and finally derive a second time, which, however, would introduce and increase noise for every step of the process.

We thus decided to model the signal as a Gaussian mixture model (GMM) of skewed generalized normal distributions because it provided a faithful mathematical representation of the signal while reducing the noise below a set threshold (Figure S1F). An advantage of the GMM is its conversion into a probability distribution (PDF^S^) and a cumulative distribution (CDF^S^) through explicit mathematical operations instead of a numerical approximation. This allowed us to summarize the difference between curves into a single value: the cumulative value at maximum variance (CMV). Under our initial assumptions about DNA states at extreme temperatures, the CDF^S^ at temperature *T* represents the probability that the reporter DNA is denatured at or below temperature *T*. This probabilistic interpretation provided our rigorous base framework for quantifying differences between samples and assessing enzymatic activity.

### 3.2 MCA Can Discriminate Cleavage

To challenge the hypothesis that melting curve analysis could be used to detect Cas9 cleavage, we compared the melting curves of reactions containing an on-target sgRNA (*RPP30 Trg1* or *SARS-CoV*-*2 E Trg1*) to reactions with a scrambled sgRNA (*Scr*) and reactions where no sgRNA was added (replaced with water).

When using the fluorophore-quencher reporter DNA *FQ*-*RPP30*, we observed a clear separation in melting peaks between on-target and control sgRNA, with *RPP30 Trg1* having a melting temperature (*T*_m_) 10 °C lower. The peaks are well resolved with the controls crossing over the on-target samples around 66 °C (Figure 1A). The statistical significance of this observation is also supported by the CMV (Figure 1B), while no significant difference was present between the controls and the Scr and water samples.

**Figure 1:**
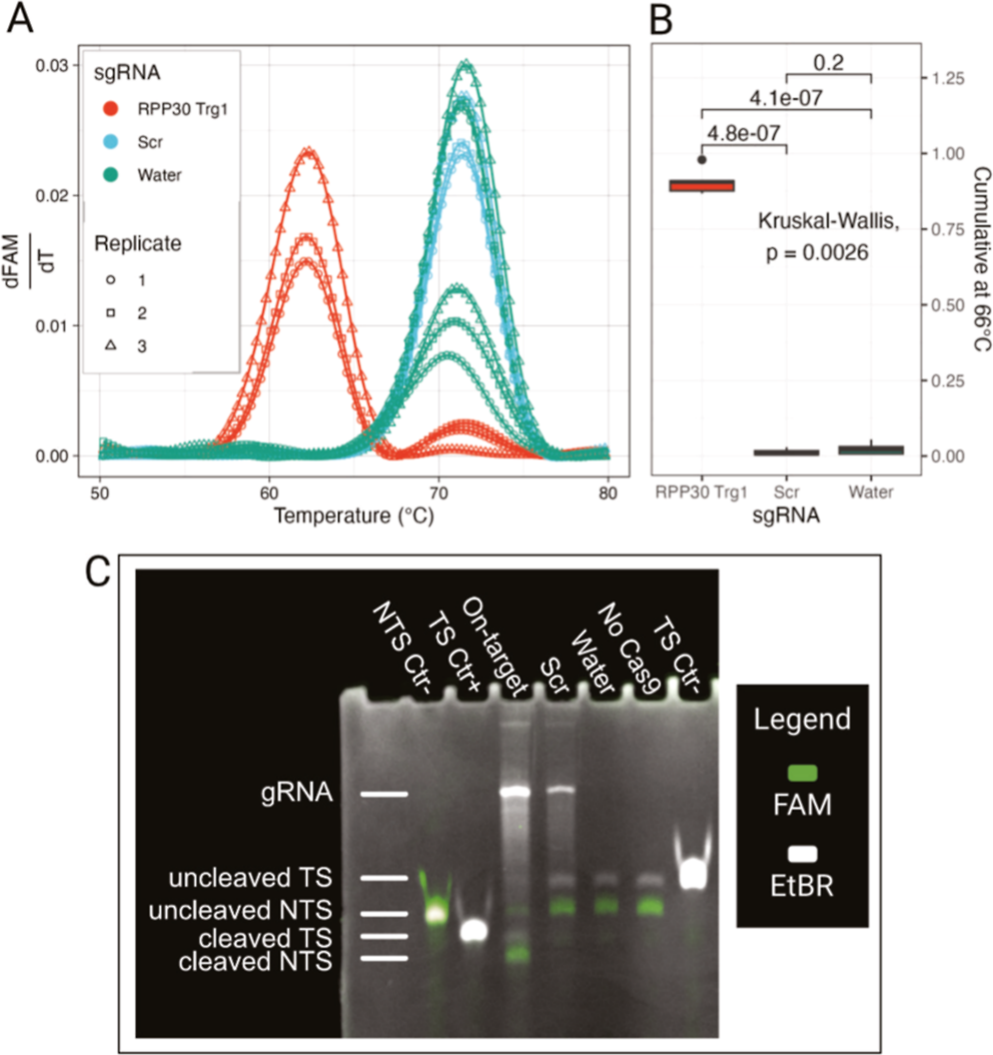
(A) Melting curve derivative and (B) cumulative at 66 °C for fluorophore-quencher reporter *FQ*-*RPP30*, showing a clear separation between the melting curves of on-target and controls sgRNA samples. (C) Denaturing PAGE in false color overlay of FAM and EtBr staining confirms the cleavage of both strands of the reporter.

SYBR Green-based reporter DNA *SYBR RPP30* shows a similar behavior, again with *RPP30 Trg1* having *T*_m_ 10 °C lower than that of the controls (Figure 2A).

**Figure 2:**
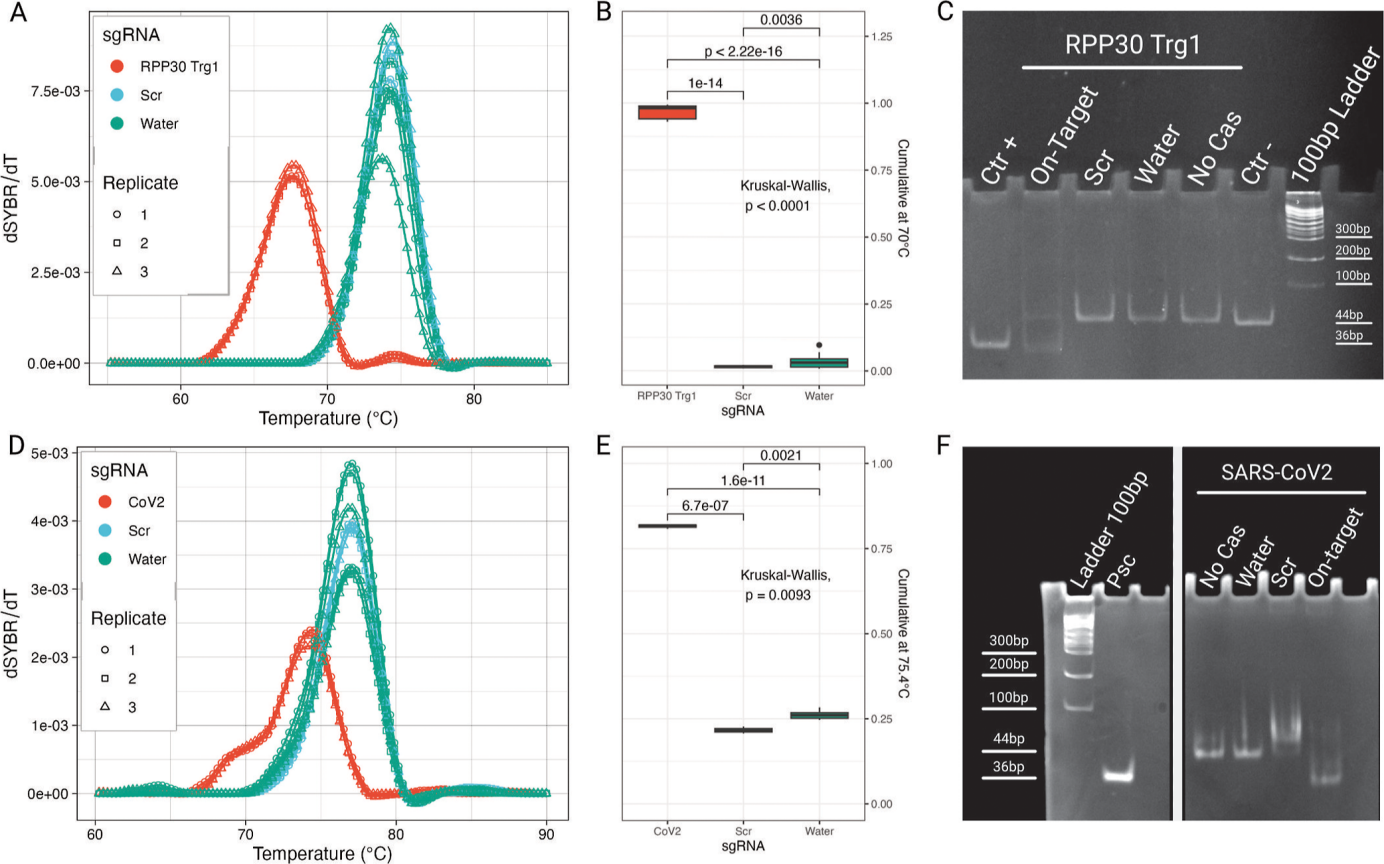
(A) Melting curve and (B) cumulative at 70 °C for *SYBR RPP30* reporter DNA, which shows the separation of the melting peaks for on-target and control sgRNA samples and (C) native PAGE, which confirms DNA cleavage. (D) Melting curve and (E) cumulative at 75.4 °C for *SYBR SARS*-*CoV*-*2* reporter DNA show a less resolute separation in melting peaks. Data analysis still supports the observed difference although at a lower significance. (F) Native PAGE confirms target cleavage.

For reporter DNA *SYBR SARS*-*CoV*-*2 E*, the separation between on-target and control sgRNA is less pronounced, with the *T*_m_ of *SARS*-*CoV*-*2 E Trg1* being only 3 °C lower than that of the controls (Figure 2D). This behavior was expected, as the reporter DNA was purposefully designed to have closer *T*_m_ values to challenge the robustness of the assay. Even though the statistical analysis supports the hypothesis that the samples treated with sgRNA *SARS*-*CoV*-*2 E Trg1* are different from the controls, the overlap between the two melting profiles impacts the specificity of the assay, reduces the statistical significance, and increases the risk of false positives.

In fact, we can give two interpretations for the value of the cumulative: either as the percentage of reporter DNA that is denatured under the temperature *T* or as the probability that the reporter DNA is fully denatured at a temperature equal to or lower than *T*.

Under this last interpretation, the value of the CMV for the controls is the false positive rate for the assay, which is around 0.25 for reporter *SYBR SARS*-*CoV*-*2 E*.

### 3.3 Electrophoretic Separation Confirms Cleavage

We validated the reporter DNA cleavage through gel electrophoresis using denaturing PAGE for the fluorophore-quencher reporter *FQ*-*RPP30* and native PAGE for the SYBR Green-based reporters *SYBR RPP30* and *SYBR SARS*-*CoV*-*2 E*.

For *FQ*-*RPP30*, we first visualized FAM fluorescence to specifically track the nontarget strand (NTS), followed by EtBr staining to reveal all nucleic acids (Figure 1C). The on-target sample exhibited two distinct lower bands corresponding to the cleaved target strand (TS) and NTS, highlighted in the false colors, confirming that both strands of the reporter have been cleaved. All control samples showed only the bands corresponding to the intact reporter DNA, demonstrating the specificity of the cleavage reaction.

Native PAGE analysis of *SYBR RPP30* and *SYBR SARS*-*CoV*-*2 E* reporters, visualized with SYBR Green I staining, revealed specific cleavage products only in the on-target samples (Figure 2C,F) and in none of the controls. Some uncleaved reporter DNA was visible in the on-target samples but not significantly detectable in the corresponding melting curve analysis (not shown). We believe this may stem from the increased concentration of glycerol (>10%) that was introduced when the reactions were scaled up, a requirement to reach the minimal concentration to visualize them on gel. Thus, while the PAGE analysis provides a qualitative confirmation of the cleavage, a more precise quantitative analysis is shown later.

### 3.4 MCA Readout Is PAM-Dependent

We considered the possibility that the change in the melting curve may be caused by an RNA:DNA hybridization of the on-target sgRNA to the reporter DNA. To check this hypothesis, we used a PAMless variant of the reporter DNA with the *RPP30* target, *SYBR RPP30 PAMless*, which should not be cleaved by the Cas9 protein.

The melting curves of the PAMless reporter show no significant difference between the on-target and control sgRNA samples (Figure 3A,B). PAGE analysis confirms the complete absence of cleavage (Figure 3C), which furthermore acts as a sanity check to ensure that the PAM specificity requirement is not influenced by the assay.

**Figure 3:**
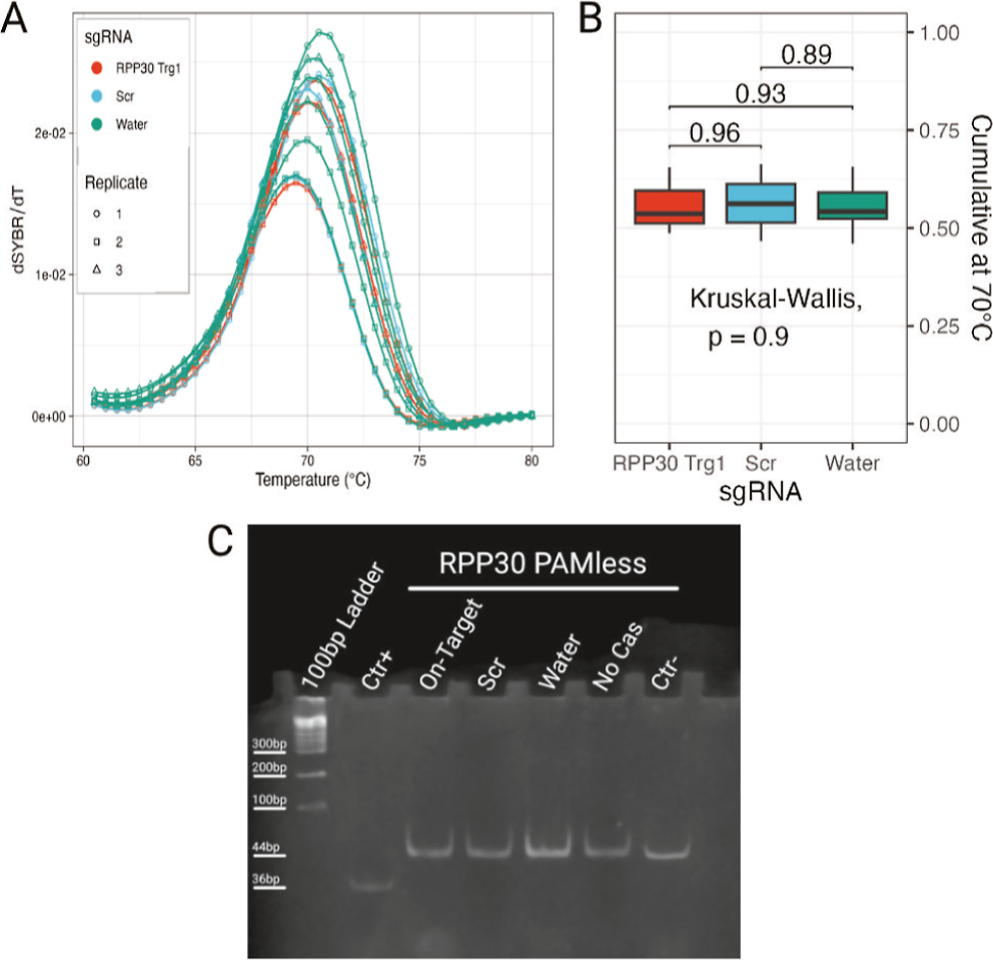
(A) Melting curve, (B) cumulative at 70 °C of PAMless RPP30 SYBR Green-based reporter, and (C) native PAGE stained with SYBR Green I. No difference is observed between on-target and control samples neither in the melting curves, cumulative value, or bands in the gel.

This also indicates that the observed difference in melting curves cannot be attributed to the hybridization of the sgRNA to the reporter DNA alone and that the cleavage activity must be responsible for it.

### 3.5 Relationship of Cleavage Quantification and MCA Signal

To quantify the relationship between the amount of cleaved reporter DNA and the signal obtained from the melting curve analysis, we prepared synthetic oligonucleotides equivalent to the products of the reaction (pseudocleaved) and mixed them with uncleaved reporter DNA in different ratios.

The relationship between the amount of pseudocleaved product and the CMV for reporter DNA *SYBR RPP30* appears to be linear (Figure 4A). When evaluating the fit quality of a *y* = *x* equation to the data—which assumes that when the sample contains only uncleaved reporter, the CMV value should be 0, and when the sample contains only cleaved reporter, the CMV value should be 1—we obtained a *R* ^2^ = 0.93 and a mean absolute error of 0.081. These results suggest that the amount of cleaved reporter DNA is directly proportional to the signal obtained from the melting curve analysis, with the real value within 10% of the result for this particular reporter DNA.

**Figure 4:**
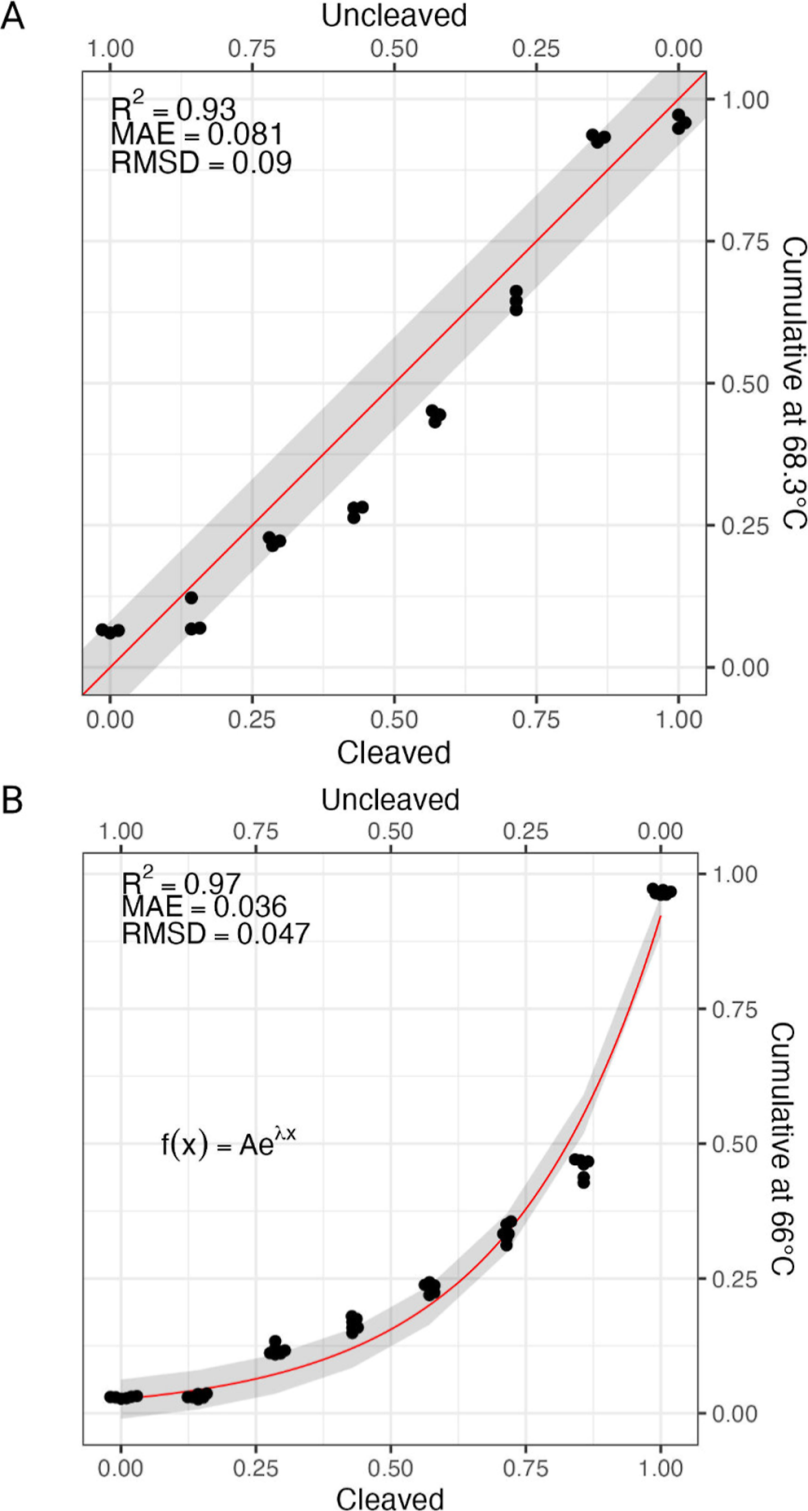
Relationship between synthetic product amount and measured cumulative at max variance. (A) For the SYBR Green-based reporter *SYBR RPP30*, this relationship is linear, (B) while in the case of fluorophore-quencher reporter *FQ*-*RPP30*, it is better explained by an exponential equation.

On the other hand, the relationship between the amount of pseudocleaved product and the CMV for reporter DNA *FQ*-*RPP30* was clearly not linear and was better explained by an exponential eq (Figure 4B). This is likely explained by a difference in the mechanism that produces the quenching effect in the uncleaved reporter and in the cleaved reporter. In the uncleaved reporter, the denaturation of DNA allows the quencher to be closer to the fluorophore, which results in a quenching effect. However, after cleavage, the quencher is separated from the fluorophore, and the quenching effect observed is caused only by the interaction of the FAM molecule with the surrounding nucleic acids,^28,29^ a phenomenon which was used in the past for melting curve analysis.^25^

### 3.6 Heat Treatment Stops Cas9 Enzymatic Activity at 53.9 °C

An important assumption of our assay with regards to the inactivation of Cas9 activity is that the denaturation step at 95 °C for 1 min is sufficient to fully stop the reaction. This is a critical step for performing accurate temporal measurements and controlling the reaction progress because it can be directly controlled by the thermal cycler without the need for external intervention.

To verify that the Cas9 protein is effectively inactivated and that the reaction cannot continue after this step, we performed heat treatments starting from 35 to 60 °C on mixtures of Cas9 protein and sgRNA before the addition of reporter DNA. The results show that the reaction is completely inactivated between 52 and 55 °C, with no recovery of activity (Figure 5A,B). To estimate a definitive inactivation temperature, we performed a logistic regression of the cumulative values at 70 °C—requiring the addition of a small amount of noise to the heat treatment value—indicating an inactivation temperature of approximately 53.9 °C (Figure 5C), which is consistent with previous reports.^11,30^ However, the exact temperature of inactivation may vary depending on the specific conditions of the reaction and, in particular, the length of the inactivation step. The described results altogether definitely support that the inactivation step at 95 °C for 1 min is fully sufficient to stop the reaction.

**Figure 5:**
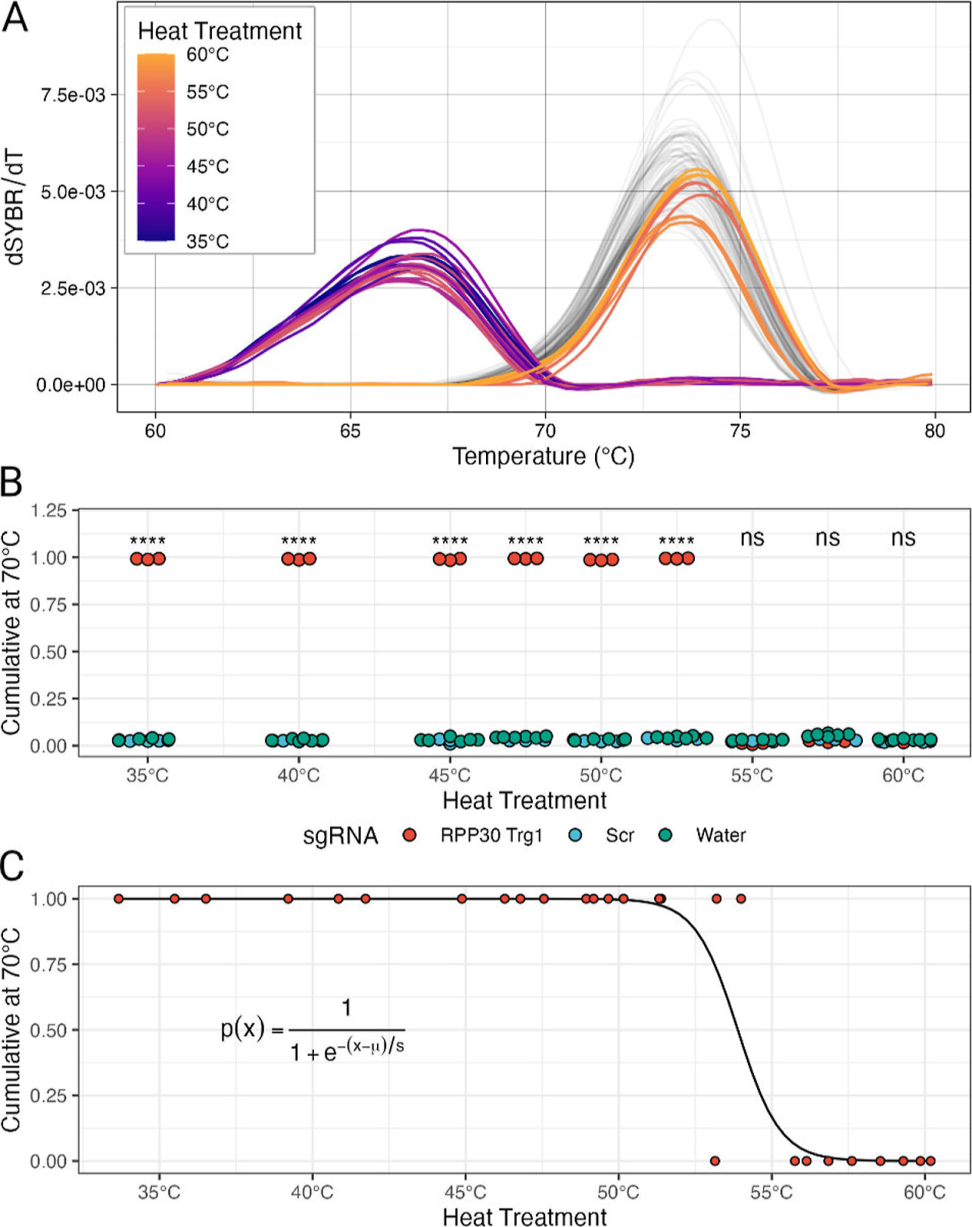
(A) Melting curves of cleavage reactions after heat treatment at different temperatures; multiple replicates, with controls colored in gray. (B) Cumulative at 70 °C of cleavage reaction after the heat treatment. A clear interruption of Cas9 activity between 52.5 and 55 °C is evident. (C) Logistic regression of cumulative values (with noise added) indicates that the inactivation temperature is approximately 53.9 °C.

### 3.7 Kinetics of the Assay

To evaluate the kinetics of the cleavage using the presented assay, we stopped the reaction at different time points and observed the change over time in the melting curve. As expected, the reaction proceeds very quickly, reaching 95% of the maximum in 140 s (2M 20S) for the SYBR Green-based reporter *SYBR RPP30* (Figure 6A) with fitted parameters *A*_1_ = 0.40, λ_1_ = 0.36 *A*_2_ = 1.06, λ_2_ = 0.025, *C* = −0.48. For the fluorophore-quencher reporter, the reaction appears slightly slower, surpassing the 95% threshold in 856 s (14M 16S) (Figure 6B) with fitted parameters *A*_1_ = 1.31, λ_1_ = 5.74×10^–5^ *A*_2_ = 0.98, λ_2_ = 9.84×10^–3^, *C* = −9.50×10^–2^.

**Figure 6:**
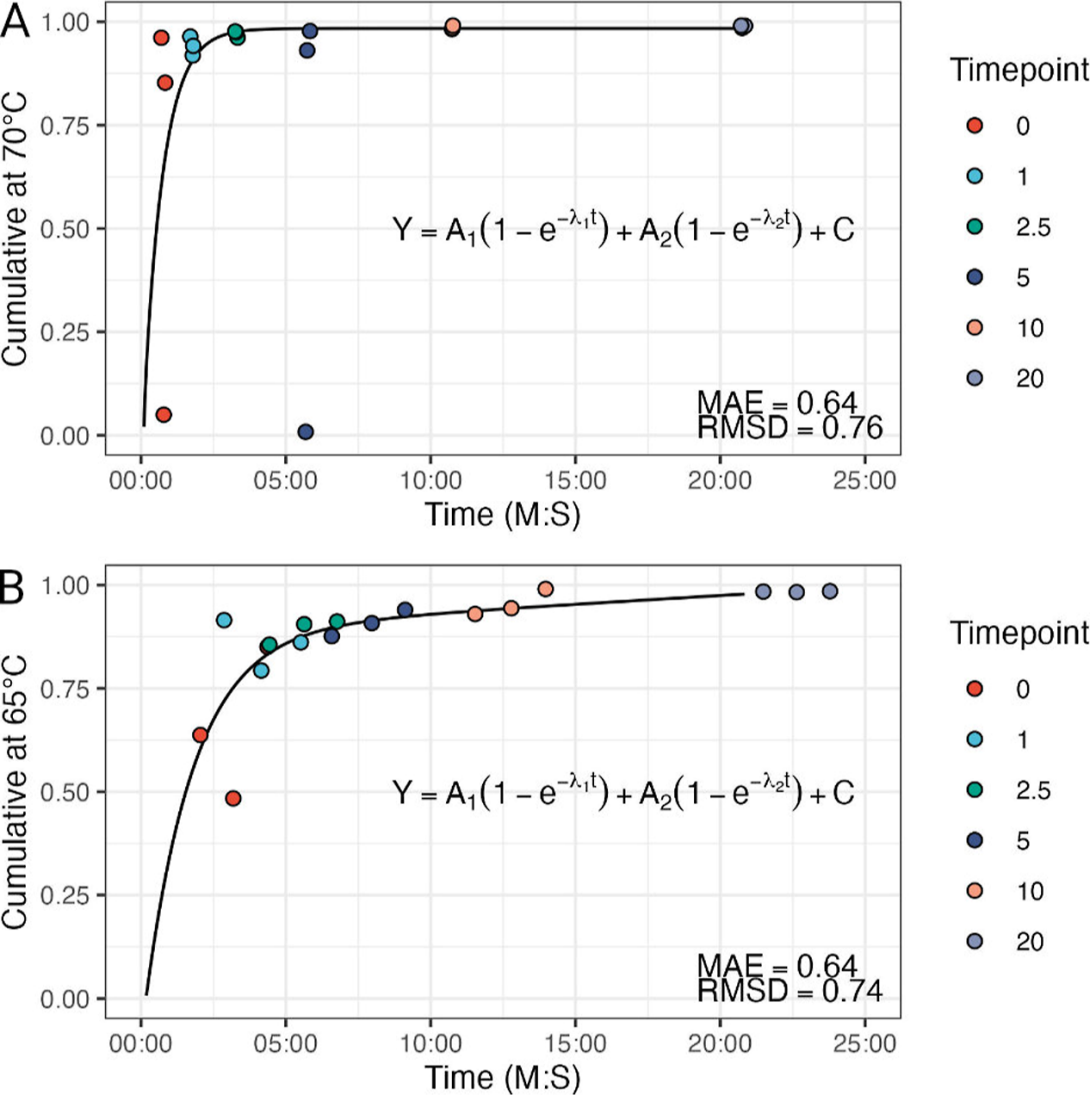
Kinetics of (A) SYBR Green-based reporter *SYBR RPP30* and (B) fluorophore-quencher reporter *FQ*-*RPP30* when sgRNA RPP30 Trg1 is used. Kinetics have been fitted with a double exponential equation. The SYBR Green-based reporter shows faster kinetics compared to the fluorophore-quencher reporter.

### 3.8 Comparing Fluo-Quench and SYBR Green-Based Reporters

In the previous sections, we have discussed two versions of the assay: one using a fluorophore-quencher based reporter and another using a SYBR Green-based reporter. While evaluating our hypothesis that MCA could be used to study Cas9 activity, we tested both reporter types for their different characteristics. Both reporters allowed us to discriminate Cas9 cleavage activity using MCA, and we consider the fluorophore-quencher-based design amenable for qualitative experiments. However, the SYBR Green-based reporter exhibited the following distinct advantages: 1. wider accessibility, utilizing standard molecular biology reagents; 2. its readout demonstrated a linear relationship between cleavage amount and signal obtained (Section 3.5); 3. faster cleavage kinetics, facilitating more rapid experimental iterations.

The primary limitation of the SYBR Green-based approach was its dependence on data processing, particularly for addressing the characteristic shoulder in the leftmost region of the melting curves that could interfere with accurate reporter state evaluation. We addressed this challenge through baseline correction algorithms and GMM modeling. The GMM approach provides a mathematical framework that transforms fluorescence data through defined analytical steps, offering advantages over the direct numerical processing of raw data. While the described final evaluation is not the only possible option, this mathematical foundation allows for a precise quantification of the melting curve characteristics, enabling the extraction of probability distributions describing the state of the reporter DNA altogether increasing robustness and reliable comparison between independent experimental runs.

## 4 Conclusions

The described assay represents a new way to study the in vitro activity of Cas9 using melting curve analysis, which we believe is a valid tool for the exploration of the in vitro activity of endonucleases of the CRISPR/Cas family because of its speed and accessibility making it a compelling alternative to more sophisticated techniques. In particular, the SYBR Green-based version of the assay employs materials that are readily available in most laboratories, making it a cost-effective and easy-to-implement method for characterizing the activity of Cas9 and other endonucleases. Given its simplicity, this technique is well-suited for high-throughput screening applications and iterative workflows, and it could fit well in semiautomatic pipelines.

The analytical pipeline that we developed addresses several technical challenges inherent to melting curve analysis. The implementation of baseline correction algorithms reduces the impact of fluorescence artifacts, while the GMM modeling approach provides a mathematical framework for evaluating changes in the DNA state. This analytical methodology enables reliable comparison between independent experimental runs by minimizing the impact of variations in experimental conditions, such as salt concentration and reagent quantities.

The provided R package *RepFluo*-*helper*’s pipeline has been configured to be open and flexible to improvements and modifications since each individual step can be replaced if required.

Although this assay was developed using *SpyCas9*, we believe it has significant potential for both high-throughput and iterative in vitro applications across a broader range of nucleases including other Cas variants and even unrelated endonucleases. Its flexibility, speed, and simplicity make it a valuable tool not only for characterizing novel nucleases under diverse conditions but also for investigating sequence specificity of isothermal amplification products,^31^ conducting off-target surveillance, and studying interactions with inhibiting or enhancing compounds.

We provide the R package *RepFluo*-*helper* freely available at https://github.com/Jocarnail/RepFluo-helper.

## Supplementary Material







## Data Availability

The data underlying this study are available in the published article and its Supporting Information.

## Notes

Example of signal processing steps from raw data to GMM; comparison of melting curves obtained using commercial vs in-house purified Cas9 protein; procedure of import of data from instruments; and comparison of raw data obtained from different instruments (PDF)
Sequences of the oligonucleotides used in this study (XLSX)
Raw data of the melting curves (ZIP)
