# Supplementary material for: RepFluo, a Fast Fluorescent In Vitro Assay of Cas9 Activity Exploiting Melting Curve Analysis
Source: ACS Omega. 2025 Nov 9;10(45):53816–26. doi: 10.1021/acsomega.5c02066 (PMC12631686; doi:10.1021/acsomega.5c02066)
Supplement: Supplementary file 1 [file ao5c02066_si_001.pdf]

RepFluo, a fast fluorescent *in vitro* assay of Cas9 activity exploiting melting curve analysis Supporting information

Filippo Fronza[*,†], Roberto Verardo[‡,¶], Claudio Schneider[†,‡]

† University of Udine, Dipartimento di Medicina (DMED), Piazzale Kolbe 1, 33100 Udine, Italy
‡ LNCIB Laboratorio Nazionale CIB, Consorzio Interuniversitario per le Biotechnologie, BIC incubatori, Via Flavia 23/1, 34148 Trieste, Italy
¶ AREA Science Park, Patriciano 99, 34149 Trieste, Italy

E-mail: filippo.fronza.o@gmail.com

Contents

1 Data processing pipeline 2
2 Purified vs commercially available protein. 2
3 Choice of sequences 3
4 Import of data from instrument 3
4.1 Import of data from BioRad CFX 3
4.2 Import of data from QuantStudio Q3 4
4.3 Comparison of BioRad CFX 96 and ThemoFisher Scientific QuantStudio Q3 4

1 Data processing pipeline

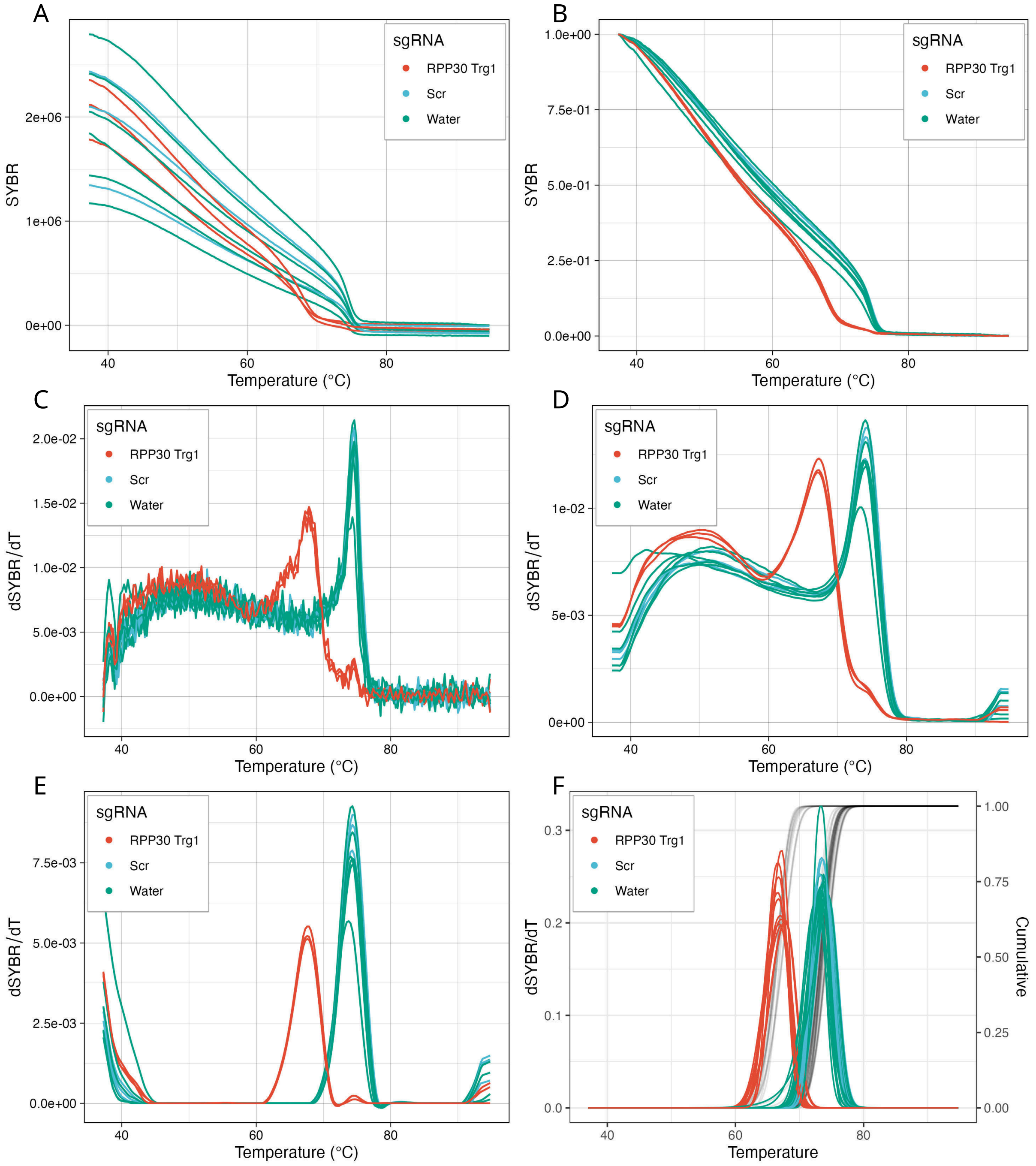

Figure S1 Processing steps applied to the raw data to obtain the final signal. (A) Raw data. (B) Min-max normalized signal. (C) Derivative without smoothing. (D) Derivative with smoothing. (E) Baseline corrected signal. (F) Representation of the Gaussian mixture model of the data, with cumulative values colored in gray

2 Purified vs commercially available protein.

We compared the effectiveness of the assay when using either a commercially available SpyCas9 protein (IDT Alt-R S.p.Cas9 Nuclease V3) or a protein purified in house. We

observed no significant difference in the assay performance. The only main difference observed was a shift in melting temperature, that can be exlained by the difference in storage buffer between the commercial and in house purified proteins.

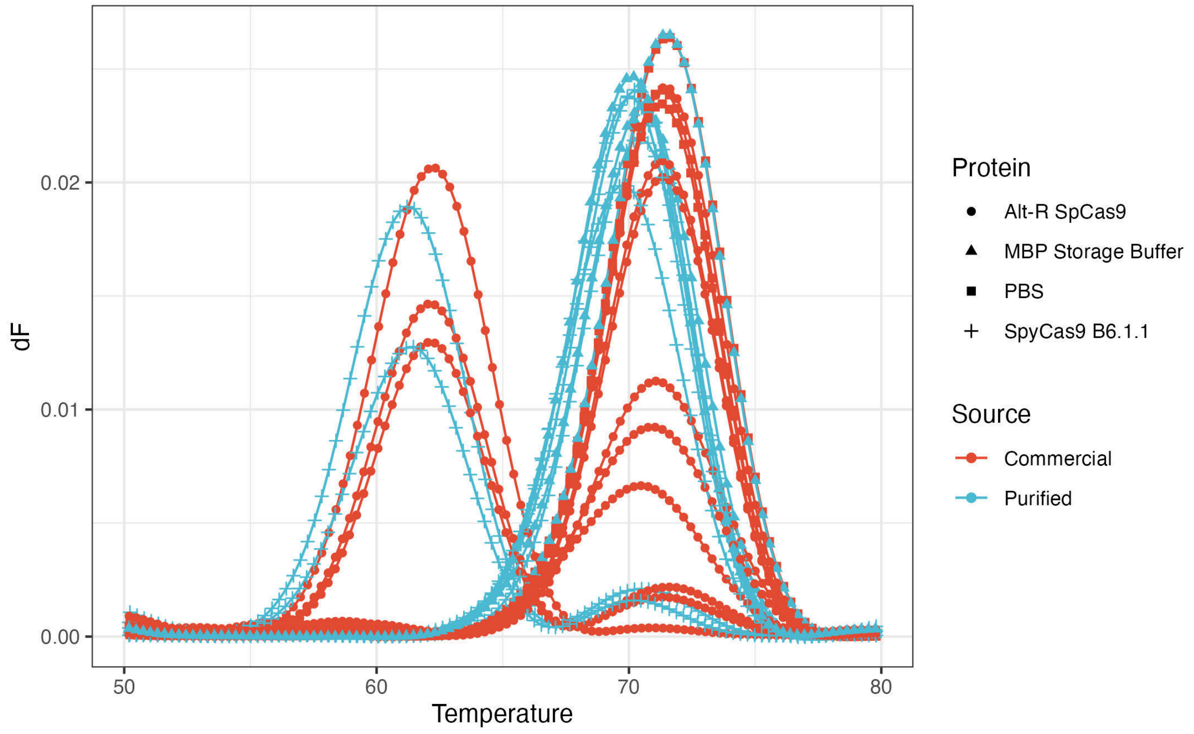

Figure S2 Comparing commercially available and purified proteins indicates no significant difference between the two, with small differences in melting temperature explained by the difference in the storage buffer used.

3 Choice of sequences

RNase P subunit RPP30 was chosen for its widespread use as an housekeeping internal control in diagnostic assays.

SARS-CoV2 gene E was selected among the relevant variant sites at the time.

4 Import of data from instrument

4.1 Import of data from BioRad CFX

Melting data was exported as csv by BioRad software. The exported data were originally organized as a table containing a different temperature for each row, a different well for each column and fluorescence readout for each cell. This data was restructured by pivot into three columns: temperature, well and fluorescent readout. Sample informations were added with a merge operation by well position before processing.

4.2 Import of data from QuantStudio Q3

Melting data was exported as excel by QuantStudio software. Melting data, originally organized as a table of Well (Well ID), Well Position (name of the well), Reading, Temperature and Fluorescence, from row 44 of the *"Melt Curve Row Data"* table were gathered, Well id information was replaced with Well Position information which was used to merge sample information onto the final dataset. Reading information and the original derivative provided by the manufacturer's software were not used.

In the RepFluo-helper package we provide helper function for this import procedures.

4.3 Comparison of BioRad CFX 96 and ThemoFisher Scientific QuantStudio Q3

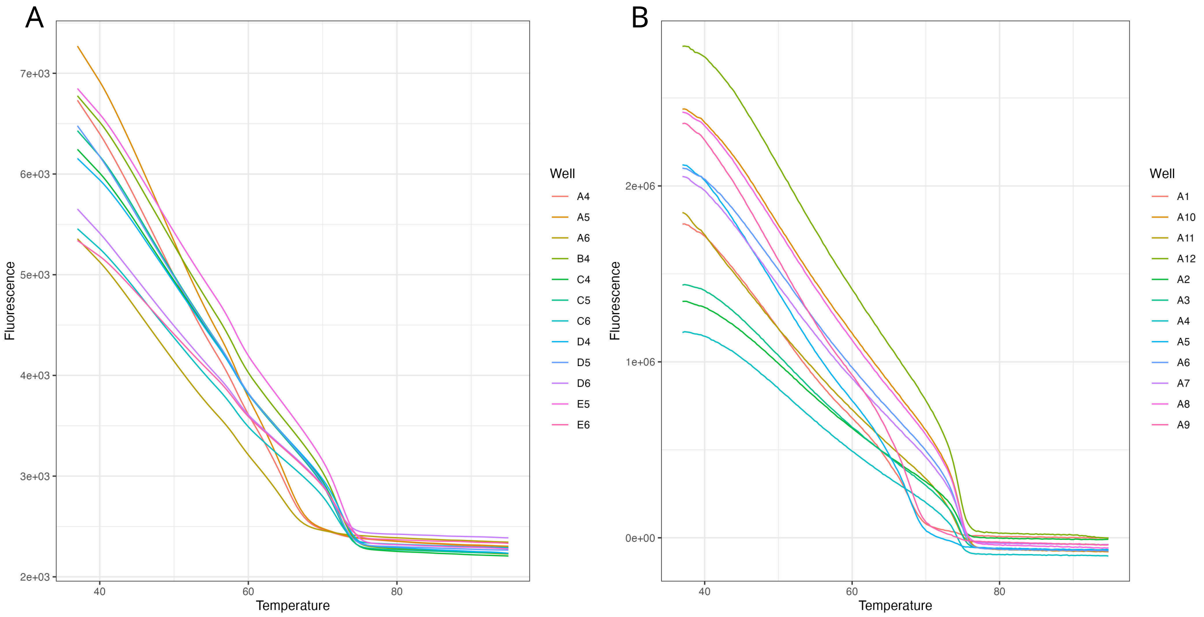

Figure S3 Example of raw melting curves obtained from BioRad CFX 96 (A) and ThermoFisher Scientific QuantStudio Q3 (B) instruments. While the range in relative fluorescence units (RFU) makes a direct comparison of the raw data obtained from the two instruments impossible, we observe the same pattern between the two, with higher variability between samples at 37°C and lower at 95°C after denaturation.
